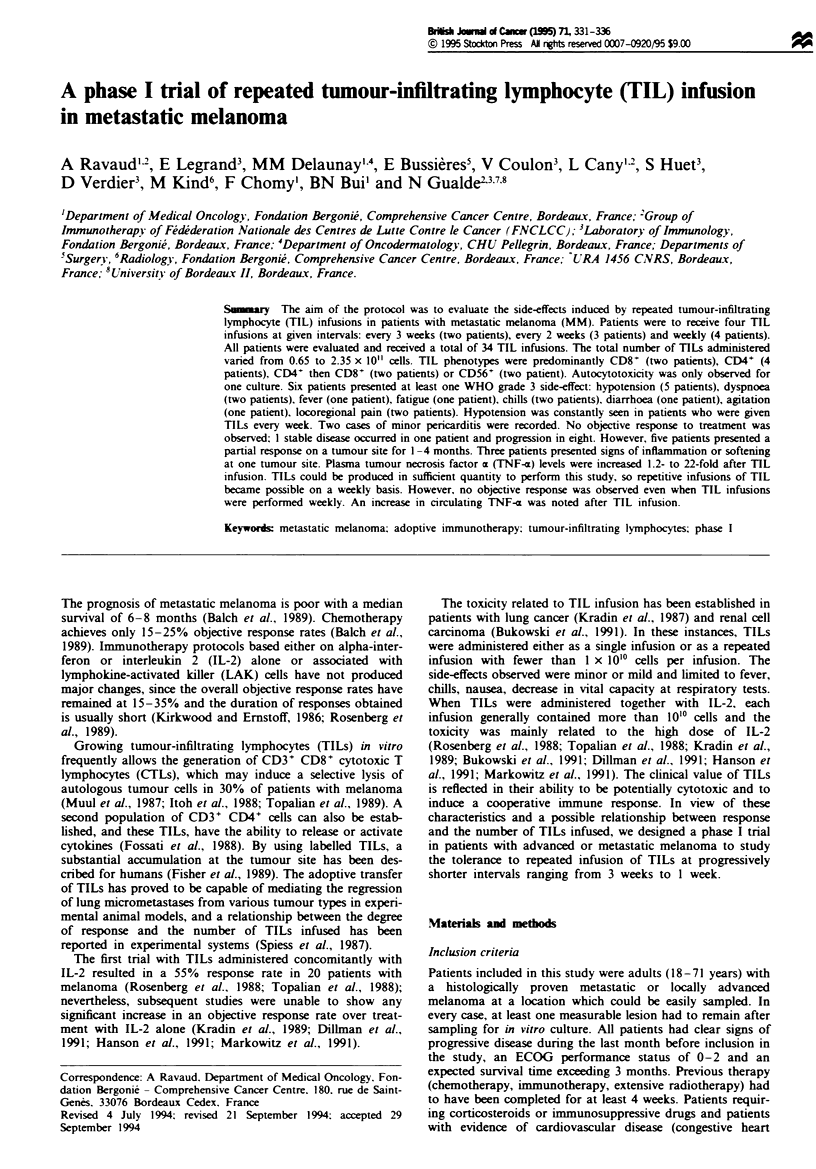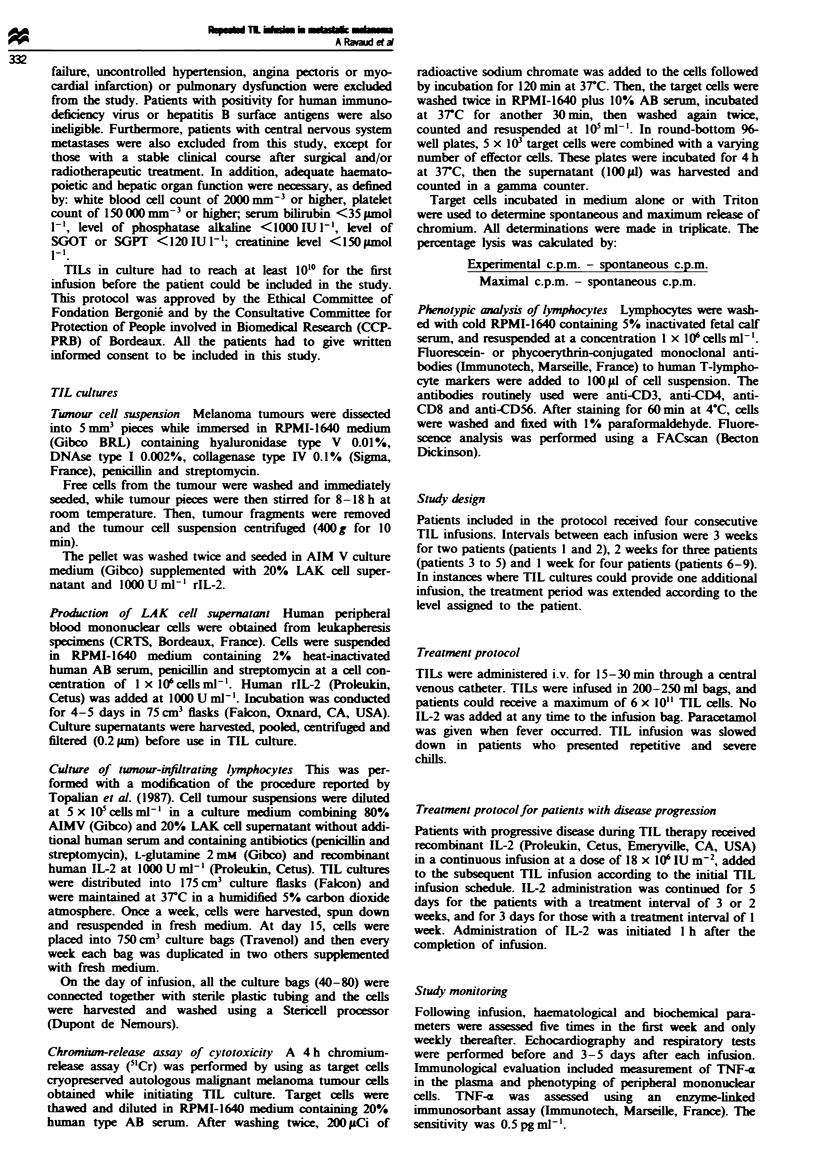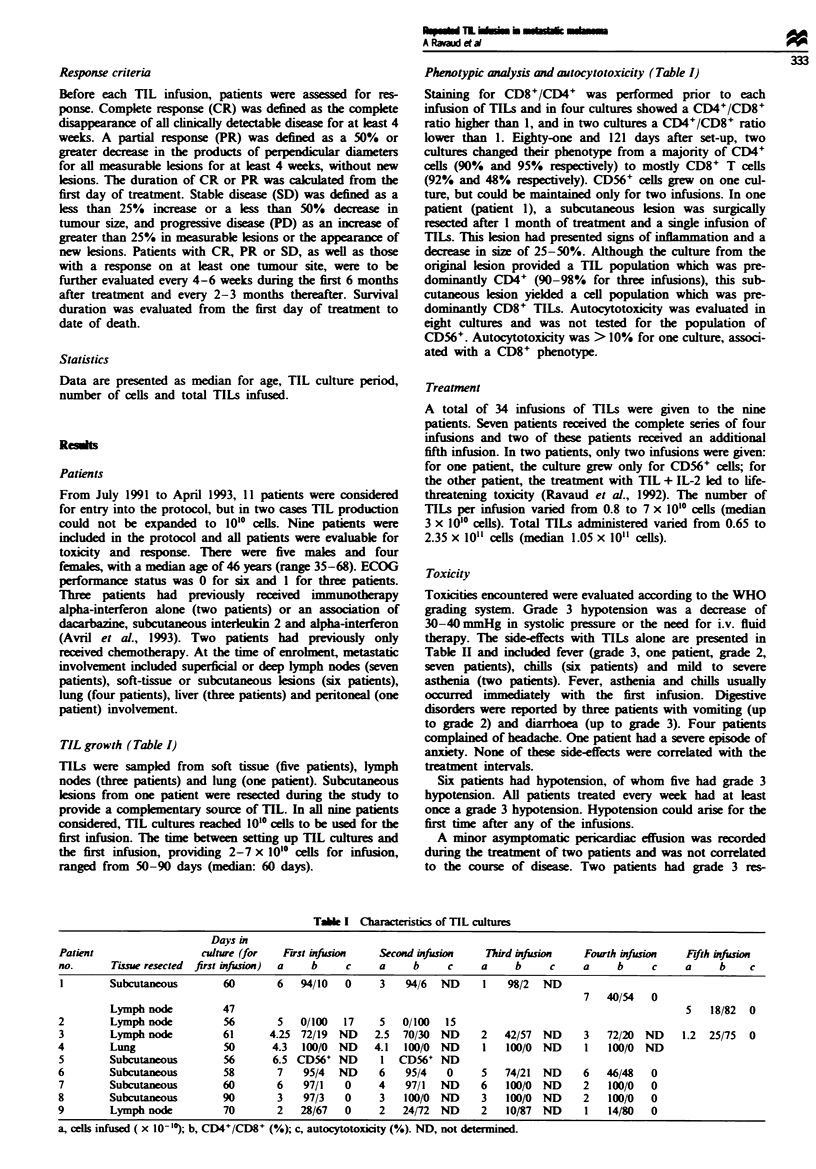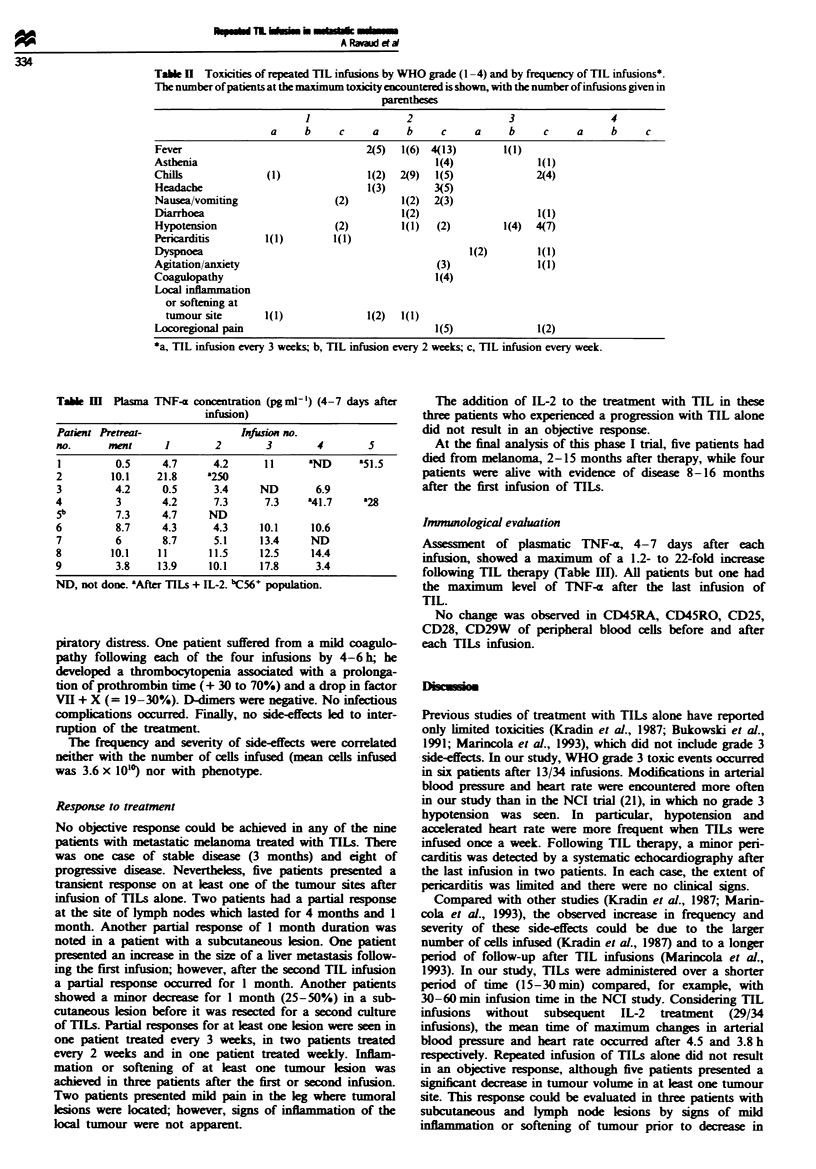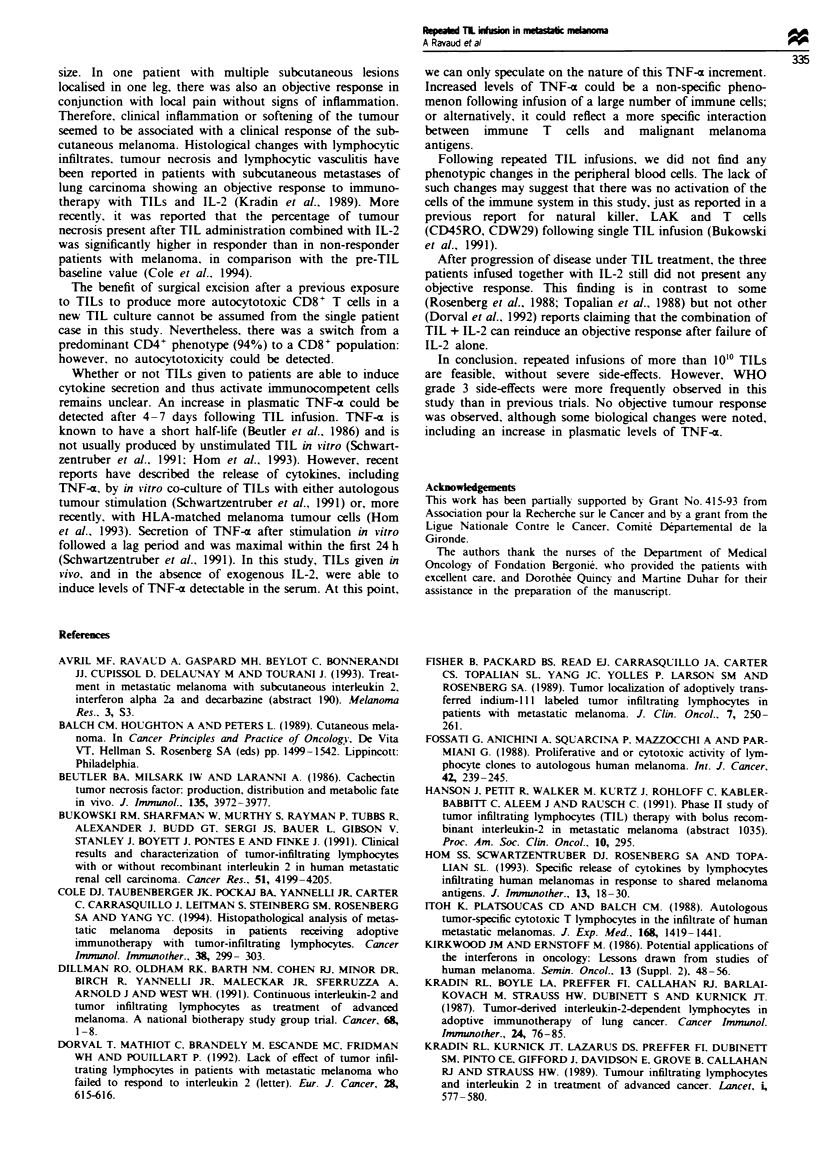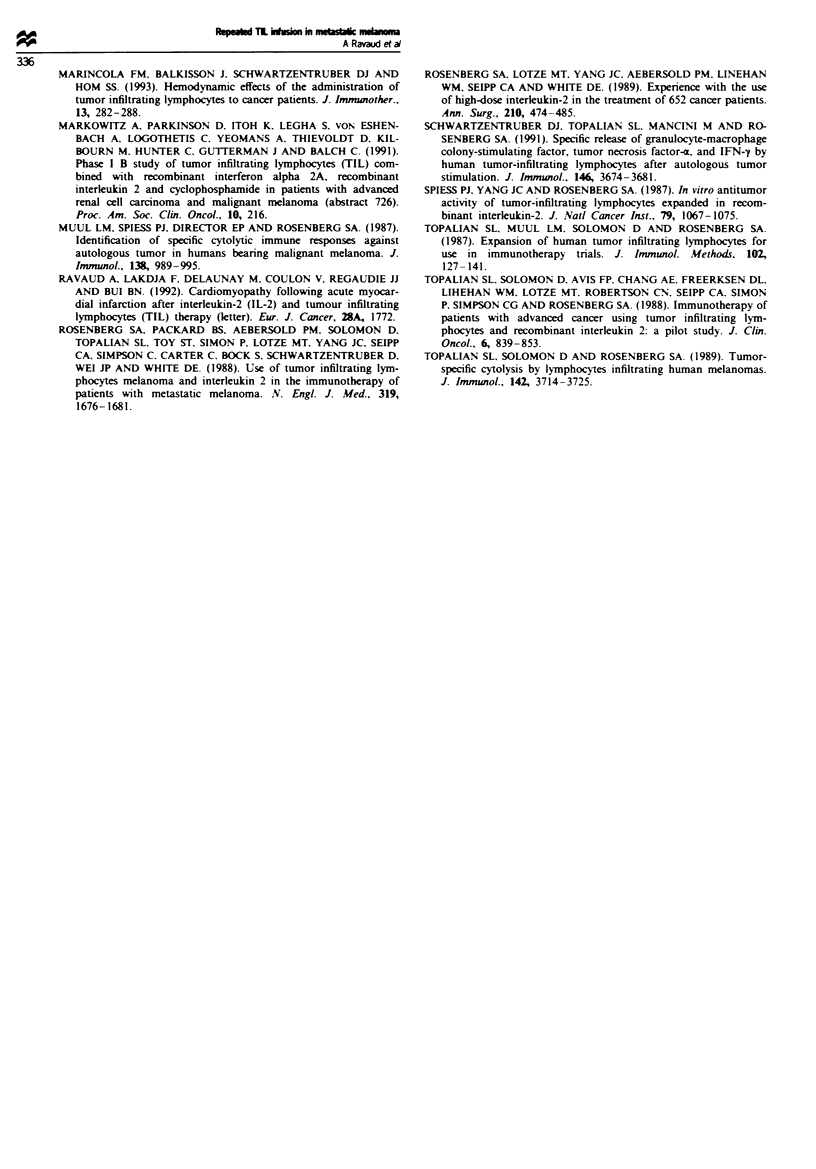# A phase I trial of repeated tumour-infiltrating lymphocyte (TIL) infusion in metastatic melanoma.

**DOI:** 10.1038/bjc.1995.66

**Published:** 1995-02

**Authors:** A. Ravaud, E. Legrand, M. M. Delaunay, E. Bussières, V. Coulon, L. Cany, S. Huet, D. Verdier, M. Kind, F. Chomy

**Affiliations:** Department of Medical Oncology, Fondation Bergonié, Comprehensive Cancer Centre, Bordeaux, France.

## Abstract

The aim of the protocol was to evaluate the side-effects induced by repeated tumour-infiltrating lymphocyte (TIL) infusions in patients with metastatic melanoma (MM). Patients were to receive four TIL infusions at given intervals: every 3 weeks (two patients), every 2 weeks (3 patients) and weekly (4 patients). All patients were evaluated and received a total of 34 TIL infusions. The total number of TILs administered varied from 0.65 to 2.34 x 10(11) cells. TIL phenotypes were predominantly CD8+ (two patients), CD4+ (4 patients), CD4+ then CD8+ (two patients) or CD56+ (two patient). Autocytotoxicity was only observed for one culture. Six patients presented at least one WHO grade 3 side-effect: hypotension (5 patients), dyspnoea (two patients), fever (one patient), fatigue (one patient), chills (two patients), diarrhoea (one patient), agitation (one patient), locoregional pain (two patients). Hypotension was constantly seen in patients who were given TILs every week. Two cases of minor pericarditis were recorded. No objective response to treatment was observed; 1 stable disease occurred in one patient and progression in eight. However, five patients presented a partial response on a tumour site for 1-4 months. Three patients presented signs of inflammation or softening at one tumour site. Plasma tumour necrosis factor alpha (TNF-alpha) levels were increased 1.2- to 22-fold after TIL infusion. TILs could be produced in sufficient quantity to perform this study, so repetitive infusions of TIL became possible on a weekly basis. However, no objective response was observed even when TIL infusions were performed weekly. An increase in circulating TNF-alpha was noted after TIL infusion.


British Journal of Cancer (1995) 71, 331-336

© 1995 Stockton Press All rights reserved 0007-0920/95 $9.00

### A phase I trial of repeated tumour-infiltrating lymphocyte (TIL) infusion in metastatic melanoma

A Ravaud[1,2], E Legrand[3], MM Delaunay[1,4], E Bussières[5], V Coulon[3], L Cany[1,2], S Huet[3], D Verdier[3], M Kind[6], F Chomy[1], BN Bui[1] and N Gualde[2,3,7,8]

[1]*Department of Medical Oncology, Fondation Bergonié, Comprehensive Cancer Centre, Bordeaux, France;* [2]*Group of Immunotherapy of Fédéderation Nationale des Centres de Lutte Contre le Cancer (FNCLCC);* [3]*Laboratory of Immunology, Fondation Bergonié, Bordeaux, France;* [4]*Department of Oncodermatology, CHU Pellegrin, Bordeaux, France; Departments of* [5]*Surgery,* [6]*Radiology, Fondation Bergonié, Comprehensive Cancer Centre, Bordeaux, France;* [7]*URA 1456 CNRS, Bordeaux, France;* [8]*University of Bordeaux II, Bordeaux, France.*

**Summary** The aim of the protocol was to evaluate the side-effects induced by repeated tumour-infiltrating lymphocyte (TIL) infusions in patients with metastatic melanoma (MM). Patients were to receive four TIL infusions at given intervals: every 3 weeks (two patients), every 2 weeks (3 patients) and weekly (4 patients). All patients were evaluated and received a total of 34 TIL infusions. The total number of TILs administered varied from 0.65 to $2.35 \times 10^{11}$ cells. TIL phenotypes were predominantly CD8$^{+}$ (two patients), CD4$^{+}$ (4 patients), CD4$^{+}$ then CD8$^{+}$ (two patients) or CD56$^{+}$ (two patient). Autocytotoxicity was only observed for one culture. Six patients presented at least one WHO grade 3 side-effect: hypotension (5 patients), dyspnoea (two patients), fever (one patient), fatigue (one patient), chills (two patients), diarrhoea (one patient), agitation (one patient), locoregional pain (two patients). Hypotension was constantly seen in patients who were given TILs every week. Two cases of minor pericarditis were recorded. No objective response to treatment was observed; 1 stable disease occurred in one patient and progression in eight. However, five patients presented a partial response on a tumour site for 1-4 months. Three patients presented signs of inflammation or softening at one tumour site. Plasma tumour necrosis factor α (TNF-α) levels were increased 1.2- to 22-fold after TIL infusion. TILs could be produced in sufficient quantity to perform this study, so repetitive infusions of TIL became possible on a weekly basis. However, no objective response was observed even when TIL infusions were performed weekly. An increase in circulating TNF-α was noted after TIL infusion.

**Keywords:** metastatic melanoma; adoptive immunotherapy; tumour-infiltrating lymphocytes; phase I

The prognosis of metastatic melanoma is poor with a median survival of 6-8 months (Balch *et al.*, 1989). Chemotherapy achieves only 15-25% objective response rates (Balch *et al.*, 1989). Immunotherapy protocols based either on alpha-interferon or interleukin 2 (IL-2) alone or associated with lymphokine-activated killer (LAK) cells have not produced major changes, since the overall objective response rates have remained at 15-35% and the duration of responses obtained is usually short (Kirkwood and Ernstoff, 1986; Rosenberg *et al.*, 1989).

Growing tumour-infiltrating lymphocytes (TILs) *in vitro* frequently allows the generation of CD3$^{+}$ CD8$^{+}$ cytotoxic T lymphocytes (CTLs), which may induce a selective lysis of autologous tumour cells in 30% of patients with melanoma (Muul *et al.*, 1987; Itoh *et al.*, 1988; Topalian *et al.*, 1989). A second population of CD3$^{+}$ CD4$^{+}$ cells can also be established, and these TILs, have the ability to release or activate cytokines (Fossati *et al.*, 1988). By using labelled TILs, a substantial accumulation at the tumour site has been described for humans (Fisher *et al.*, 1989). The adoptive transfer of TILs has proved to be capable of mediating the regression of lung micrometastases from various tumour types in experimental animal models, and a relationship between the degree of response and the number of TILs infused has been reported in experimental systems (Spiess *et al.*, 1987).

The first trial with TILs administered concomitantly with IL-2 resulted in a 55% response rate in 20 patients with melanoma (Rosenberg *et al.*, 1988; Topalian *et al.*, 1988); nevertheless, subsequent studies were unable to show any significant increase in an objective response rate over treatment with IL-2 alone (Kradin *et al.*, 1989; Dillman *et al.*, 1991; Hanson *et al.*, 1991; Markowitz *et al.*, 1991).

Correspondence: A Ravaud, Department of Medical Oncology, Fondation Bergonié - Comprehensive Cancer Centre, 180, rue de Saint-Genès, 33076 Bordeaux Cedex, France
Revised 4 July 1994; revised 21 September 1994; accepted 29 September 1994

The toxicity related to TIL infusion has been established in patients with lung cancer (Kradin *et al.*, 1987) and renal cell carcinoma (Bukowski *et al.*, 1991). In these instances, TILs were administered either as a single infusion or as a repeated infusion with fewer than $1 \times 10^{10}$ cells per infusion. The side-effects observed were minor or mild and limited to fever, chills, nausea, decrease in vital capacity at respiratory tests. When TILs were administered together with IL-2, each infusion generally contained more than $10^{10}$ cells and the toxicity was mainly related to the high dose of IL-2 (Rosenberg *et al.*, 1988; Topalian *et al.*, 1988; Kradin *et al.*, 1989; Bukowski *et al.*, 1991; Dillman *et al.*, 1991; Hanson *et al.*, 1991; Markowitz *et al.*, 1991). The clinical value of TILs is reflected in their ability to be potentially cytotoxic and to induce a cooperative immune response. In view of these characteristics and a possible relationship between response and the number of TILs infused, we designed a phase I trial in patients with advanced or metastatic melanoma to study the tolerance to repeated infusion of TILs at progressively shorter intervals ranging from 3 weeks to 1 week.

### Materials and methods

### *Inclusion criteria*

Patients included in this study were adults (18-71 years) with a histologically proven metastatic or locally advanced melanoma at a location which could be easily sampled. In every case, at least one measurable lesion had to remain after sampling for *in vitro* culture. All patients had clear signs of progressive disease during the last month before inclusion in the study, an ECOG performance status of 0-2 and an expected survival time exceeding 3 months. Previous therapy (chemotherapy, immunotherapy, extensive radiotherapy) had to have been completed for at least 4 weeks. Patients requiring corticosteroids or immunosuppressive drugs and patients with evidence of cardiovascular disease (congestive heart failure, uncontrolled hypertension, angina pectoris or myocardial infarction) or pulmonary dysfunction were excluded from the study. Patients with positivity for human immunodeficiency virus or hepatitis B surface antigens were also ineligible. Furthermore, patients with central nervous system metastases were also excluded from this study, except for those with a stable clinical course after surgical and or radiotherapeutic treatment. In addition, adequate haematopoietic and hepatic organ function were necessary, as defined by: white blood cell count of 2000 $mm^{-3}$ or higher, platelet count of 150 000 $mm^{-3}$ or higher; serum bilirubin <35 μmol $l^{-1}$, level of phosphatase alkaline <1000 IU $l^{-1}$, level of SGOT or SGPT <120 IU $l^{-1}$; creatinine level <150 μmol $l^{-1}$.

TILs in culture had to reach at least $10^{10}$ for the first infusion before the patient could be included in the study. This protocol was approved by the Ethical Committee of Fondation Bergonié and by the Consultative Committee for Protection of People involved in Biomedical Research (CCP-PRB) of Bordeaux. All the patients had to give written informed consent to be included in this study.

### TIL cultures

*Tumour cell suspension* Melanoma tumours were dissected into 5 $mm^3$ pieces while immersed in RPMI-1640 medium (Gibco BRL) containing hyaluronidase type V 0.01%, DNAse type I 0.002%, collagenase type IV 0.1% (Sigma, France), penicillin and streptomycin.

Free cells from the tumour were washed and immediately seeded, while tumour pieces were then stirred for 8–18 h at room temperature. Then, tumour fragments were removed and the tumour cell suspension centrifuged (400 *g* for 10 min).

The pellet was washed twice and seeded in AIM V culture medium (Gibco) supplemented with 20% LAK cell supernatant and 1000 U $ml^{-1}$ rIL-2.

*Production of LAK cell supernatant* Human peripheral blood mononuclear cells were obtained from leukapheresis specimens (CRTS, Bordeaux, France). Cells were suspended in RPMI-1640 medium containing 2% heat-inactivated human AB serum, penicillin and streptomycin at a cell concentration of $1 \times 10^6$ cells $ml^{-1}$. Human rIL-2 (Proleukin, Cetus) was added at 1000 U $ml^{-1}$. Incubation was conducted for 4–5 days in 75 $cm^3$ flasks (Falcon, Oxnard, CA, USA). Culture supernatants were harvested, pooled, centrifuged and filtered (0.2 μm) before use in TIL culture.

*Culture of tumour-infiltrating lymphocytes* This was performed with a modification of the procedure reported by Topalian *et al.* (1987). Cell tumour suspensions were diluted at $5 \times 10^5$ cells $ml^{-1}$ in a culture medium combining 80% AIMV (Gibco) and 20% LAK cell supernatant without additional human serum and containing antibiotics (penicillin and streptomycin), L-glutamine 2 mM (Gibco) and recombinant human IL-2 at 1000 U $ml^{-1}$ (Proleukin, Cetus). TIL cultures were distributed into 175 $cm^3$ culture flasks (Falcon) and were maintained at 37°C in a humidified 5% carbon dioxide atmosphere. Once a week, cells were harvested, spun down and resuspended in fresh medium. At day 15, cells were placed into 750 $cm^3$ culture bags (Travenol) and then every week each bag was duplicated in two others supplemented with fresh medium.

On the day of infusion, all the culture bags (40–80) were connected together with sterile plastic tubing and the cells were harvested and washed using a Stericell processor (Dupont de Nemours).

*Chromium-release assay of cytotoxicity* A 4 h chromium-release assay ($^{51}Cr$) was performed by using as target cells cryopreserved autologous malignant melanoma tumour cells obtained while initiating TIL culture. Target cells were thawed and diluted in RPMI-1640 medium containing 20% human type AB serum. After washing twice, 200 μCi of radioactive sodium chromate was added to the cells followed by incubation for 120 min at 37°C. Then, the target cells were washed twice in RPMI-1640 plus 10% AB serum, incubated at 37°C for another 30 min, then washed again twice, counted and resuspended at $10^5$ $ml^{-1}$. In round-bottom 96-well plates, $5 \times 10^3$ target cells were combined with a varying number of effector cells. These plates were incubated for 4 h at 37°C, then the supernatant (100 μl) was harvested and counted in a gamma counter.

Target cells incubated in medium alone or with Triton were used to determine spontaneous and maximum release of chromium. All determinations were made in triplicate. The percentage lysis was calculated by:

$$\frac{\text{Experimental c.p.m.} - \text{spontaneous c.p.m.}}{\text{Maximal c.p.m.} - \text{spontaneous c.p.m.}}$$

*Phenotypic analysis of lymphocytes* Lymphocytes were washed with cold RPMI-1640 containing 5% inactivated fetal calf serum, and resuspended at a concentration $1 \times 10^6$ cells $ml^{-1}$. Fluorescein- or phycoerythrin-conjugated monoclonal antibodies (Immunotech, Marseille, France) to human T-lymphocyte markers were added to 100 μl of cell suspension. The antibodies routinely used were anti-CD3, anti-CD4, anti-CD8 and anti-CD56. After staining for 60 min at 4°C, cells were washed and fixed with 1% paraformaldehyde. Fluorescence analysis was performed using a FACscan (Becton Dickinson).

### Study design

Patients included in the protocol received four consecutive TIL infusions. Intervals between each infusion were 3 weeks for two patients (patients 1 and 2), 2 weeks for three patients (patients 3 to 5) and 1 week for four patients (patients 6–9). In instances where TIL cultures could provide one additional infusion, the treatment period was extended according to the level assigned to the patient.

### Treatment protocol

TILs were administered i.v. for 15–30 min through a central venous catheter. TILs were infused in 200–250 ml bags, and patients could receive a maximum of $6 \times 10^{11}$ TIL cells. No IL-2 was added at any time to the infusion bag. Paracetamol was given when fever occurred. TIL infusion was slowed down in patients who presented repetitive and severe chills.

### Treatment protocol for patients with disease progression

Patients with progressive disease during TIL therapy received recombinant IL-2 (Proleukin, Cetus, Emeryville, CA, USA) in a continuous infusion at a dose of $18 \times 10^6$ IU $m^{-2}$, added to the subsequent TIL infusion according to the initial TIL infusion schedule. IL-2 administration was continued for 5 days for the patients with a treatment interval of 3 or 2 weeks, and for 3 days for those with a treatment interval of 1 week. Administration of IL-2 was initiated 1 h after the completion of infusion.

### Study monitoring

Following infusion, haematological and biochemical parameters were assessed five times in the first week and only weekly thereafter. Echocardiography and respiratory tests were performed before and 3–5 days after each infusion. Immunological evaluation included measurement of TNF-α in the plasma and phenotyping of peripheral mononuclear cells. TNF-α was assessed using an enzyme-linked immunosorbant assay (Immunotech, Marseille, France). The sensitivity was 0.5 pg $ml^{-1}$.

*Response criteria*

Before each TIL infusion, patients were assessed for response. Complete response (CR) was defined as the complete disappearance of all clinically detectable disease for at least 4 weeks. A partial response (PR) was defined as a 50% or greater decrease in the products of perpendicular diameters for all measurable lesions for at least 4 weeks, without new lesions. The duration of CR or PR was calculated from the first day of treatment. Stable disease (SD) was defined as a less than 25% increase or a less than 50% decrease in tumour size, and progressive disease (PD) as an increase of greater than 25% in measurable lesions or the appearance of new lesions. Patients with CR, PR or SD, as well as those with a response on at least one tumour site, were to be further evaluated every 4–6 weeks during the first 6 months after treatment and every 2–3 months thereafter. Survival duration was evaluated from the first day of treatment to date of death.

*Statistics*

Data are presented as median for age, TIL culture period, number of cells and total TILs infused.

### Results

*Patients*

From July 1991 to April 1993, 11 patients were considered for entry into the protocol, but in two cases TIL production could not be expanded to $10^{10}$ cells. Nine patients were included in the protocol and all patients were evaluable for toxicity and response. There were five males and four females, with a median age of 46 years (range 35–68). ECOG performance status was 0 for six and 1 for three patients. Three patients had previously received immunotherapy alpha-interferon alone (two patients) or an association of dacarbazine, subcutaneous interleukin 2 and alpha-interferon (Avril *et al.*, 1993). Two patients had previously only received chemotherapy. At the time of enrolment, metastatic involvement included superficial or deep lymph nodes (seven patients), soft-tissue or subcutaneous lesions (six patients), lung (four patients), liver (three patients) and peritoneal (one patient) involvement.

*TIL growth (Table I)*

TILs were sampled from soft tissue (five patients), lymph nodes (three patients) and lung (one patient). Subcutaneous lesions from one patient were resected during the study to provide a complementary source of TIL. In all nine patients considered, TIL cultures reached $10^{10}$ cells to be used for the first infusion. The time between setting up TIL cultures and the first infusion, providing $2-7 \times 10^{10}$ cells for infusion, ranged from 50–90 days (median: 60 days).

*Phenotypic analysis and autocytotoxicity (Table I)*

Staining for $CD8^+/CD4^+$ was performed prior to each infusion of TILs and in four cultures showed a $CD4^+/CD8^+$ ratio higher than 1, and in two cultures a $CD4^+/CD8^+$ ratio lower than 1. Eighty-one and 121 days after set-up, two cultures changed their phenotype from a majority of $CD4^+$ cells (90% and 95% respectively) to mostly $CD8^+$ T cells (92% and 48% respectively). $CD56^+$ cells grew on one culture, but could be maintained only for two infusions. In one patient (patient 1), a subcutaneous lesion was surgically resected after 1 month of treatment and a single infusion of TILs. This lesion had presented signs of inflammation and a decrease in size of 25–50%. Although the culture from the original lesion provided a TIL population which was predominantly $CD4^+$ (90–98% for three infusions), this subcutaneous lesion yielded a cell population which was predominantly $CD8^+$ TILs. Autocytotoxicity was evaluated in eight cultures and was not tested for the population of $CD56^+$. Autocytotoxicity was >10% for one culture, associated with a $CD8^+$ phenotype.

*Treatment*

A total of 34 infusions of TILs were given to the nine patients. Seven patients received the complete series of four infusions and two of these patients received an additional fifth infusion. In two patients, only two infusions were given: for one patient, the culture grew only for $CD56^+$ cells; for the other patient, the treatment with TIL + IL-2 led to life-threatening toxicity (Ravaud *et al.*, 1992). The number of TILs per infusion varied from 0.8 to $7 \times 10^{10}$ cells (median $3 \times 10^{10}$ cells). Total TILs administered varied from 0.65 to $2.35 \times 10^{11}$ cells (median $1.05 \times 10^{11}$ cells).

*Toxicity*

Toxicities encountered were evaluated according to the WHO grading system. Grade 3 hypotension was a decrease of 30–40 mmHg in systolic pressure or the need for i.v. fluid therapy. The side-effects with TILs alone are presented in Table II and included fever (grade 3, one patient, grade 2, seven patients), chills (six patients) and mild to severe asthenia (two patients). Fever, asthenia and chills usually occurred immediately with the first infusion. Digestive disorders were reported by three patients with vomiting (up to grade 2) and diarrhoea (up to grade 3). Four patients complained of headache. One patient had a severe episode of anxiety. None of these side-effects were correlated with the treatment intervals.

Six patients had hypotension, of whom five had grade 3 hypotension. All patients treated every week had at least once a grade 3 hypotension. Hypotension could arise for the first time after any of the infusions.

A minor asymptomatic pericardiac effusion was recorded during the treatment of two patients and was not correlated to the course of disease. Two patients had grade 3 res-

**Table I** Characteristics of TIL cultures

| Patient no. | Tissue resected | Days in culture (for first infusion) | First infusion a | b | c | Second infusion a | b | c | Third infusion a | b | c | Fourth infusion a | b | c | Fifth infusion a | b | c |
|---|---|---|---|---|---|---|---|---|---|---|---|---|---|---|---|---|---|
| 1 | Subcutaneous | 60 | 6 | 94/10 | 0 | 3 | 94/6 | ND | 1 | 98/2 | ND | | | | | | |
| | | | | | | | | | | | | 7 | 40/54 | 0 | | | |
| | Lymph node | 47 | | | | | | | | | | | | | 5 | 18/82 | 0 |
| 2 | Lymph node | 56 | 5 | 0/100 | 17 | 5 | 0/100 | 15 | | | | | | | | | |
| 3 | Lymph node | 61 | 4.25 | 72/19 | ND | 2.5 | 70/30 | ND | 2 | 42/57 | ND | 3 | 72/20 | ND | 1.2 | 25/75 | 0 |
| 4 | Lung | 50 | 4.3 | 100/0 | ND | 4.1 | 100/0 | ND | 1 | 100/0 | ND | 1 | 100/0 | ND | | | |
| 5 | Subcutaneous | 56 | 6.5 | $CD56^+$ | ND | 1 | $CD56^+$ | ND | | | | | | | | | |
| 6 | Subcutaneous | 58 | 7 | 95/4 | ND | 6 | 95/4 | 0 | 5 | 74/21 | ND | 6 | 46/48 | 0 | | | |
| 7 | Subcutaneous | 60 | 6 | 97/1 | 0 | 4 | 97/1 | ND | 6 | 100/0 | ND | 2 | 100/0 | 0 | | | |
| 8 | Subcutaneous | 90 | 3 | 97/3 | 0 | 3 | 100/0 | ND | 3 | 100/0 | ND | 2 | 100/0 | 0 | | | |
| 9 | Lymph node | 70 | 2 | 28/67 | 0 | 2 | 24/72 | ND | 2 | 10/87 | ND | 1 | 14/80 | 0 | | | |

a, cells infused ($\times 10^{-10}$); b, $CD4^+/CD8^+$ (%); c, autocytotoxicity (%). ND, not determined.

**Table II** Toxicities of repeated TIL infusions by WHO grade (1–4) and by frequency of TIL infusions*. The number of patients at the maximum toxicity encountered is shown, with the number of infusions given in parentheses

| | *1* | | | *2* | | | *3* | | | *4* | | |
|---|---|---|---|---|---|---|---|---|---|---|---|---|
| | *a* | *b* | *c* | *a* | *b* | *c* | *a* | *b* | *c* | *a* | *b* | *c* |
| Fever | | | | 2(5) | 1(6) | 4(13) | | 1(1) | | | | |
| Asthenia | | | | | | 1(4) | | | 1(1) | | | |
| Chills | (1) | | | 1(2) | 2(9) | 1(5) | | | 2(4) | | | |
| Headache | | | | 1(3) | | 3(5) | | | | | | |
| Nausea/vomiting | | | (2) | | 1(2) | 2(3) | | | | | | |
| Diarrhoea | | | | | 1(2) | | | | 1(1) | | | |
| Hypotension | | | (2) | | 1(1) | (2) | | 1(4) | 4(7) | | | |
| Pericarditis | 1(1) | | 1(1) | | | | | | | | | |
| Dyspnoea | | | | | | | 1(2) | | 1(1) | | | |
| Agitation/anxiety | | | | | | (3) | | | 1(1) | | | |
| Coagulopathy | | | | | | 1(4) | | | | | | |
| Local inflammation or softening at tumour site | 1(1) | | | 1(2) | 1(1) | | | | | | | |
| Locoregional pain | | | | | | 1(5) | | | 1(2) | | | |

*a, TIL infusion every 3 weeks; b, TIL infusion every 2 weeks; c, TIL infusion every week.

**Table III** Plasma TNF-α concentration ($pg\,ml^{-1}$) (4–7 days after infusion)

| *Patient no.* | *Pretreatment* | *Infusion no.* 1 | 2 | 3 | 4 | 5 |
|---|---|---|---|---|---|---|
| 1 | 0.5 | 4.7 | 4.2 | 11 | [a]ND | [a]51.5 |
| 2 | 10.1 | 21.8 | [a]250 | | | |
| 3 | 4.2 | 0.5 | 3.4 | ND | 6.9 | |
| 4 | 3 | 4.2 | 7.3 | 7.3 | [a]41.7 | [a]28 |
| 5[b] | 7.3 | 4.7 | ND | | | |
| 6 | 8.7 | 4.3 | 4.3 | 10.1 | 10.6 | |
| 7 | 6 | 8.7 | 5.1 | 13.4 | ND | |
| 8 | 10.1 | 11 | 11.5 | 12.5 | 14.4 | |
| 9 | 3.8 | 13.9 | 10.1 | 17.8 | 3.4 | |

ND, not done. [a]After TILs + IL-2. [b]$C56^+$ population.

piratory distress. One patient suffered from a mild coagulopathy following each of the four infusions by 4–6 h; he developed a thrombocytopenia associated with a prolongation of prothrombin time (+ 30 to 70%) and a drop in factor VII + X (= 19–30%). D-dimers were negative. No infectious complications occurred. Finally, no side-effects led to interruption of the treatment.

The frequency and severity of side-effects were correlated neither with the number of cells infused (mean cells infused was $3.6 \times 10^{10}$) nor with phenotype.

### *Response to treatment*

No objective response could be achieved in any of the nine patients with metastatic melanoma treated with TILs. There was one case of stable disease (3 months) and eight of progressive disease. Nevertheless, five patients presented a transient response on at least one of the tumour sites after infusion of TILs alone. Two patients had a partial response at the site of lymph nodes which lasted for 4 months and 1 month. Another partial response of 1 month duration was noted in a patient with a subcutaneous lesion. One patient presented an increase in the size of a liver metastasis following the first infusion; however, after the second TIL infusion a partial response occurred for 1 month. Another patients showed a minor decrease for 1 month (25–50%) in a subcutaneous lesion before it was resected for a second culture of TILs. Partial responses for at least one lesion were seen in one patient treated every 3 weeks, in two patients treated every 2 weeks and in one patient treated weekly. Inflammation or softening of at least one tumour lesion was achieved in three patients after the first or second infusion. Two patients presented mild pain in the leg where tumoral lesions were located; however, signs of inflammation of the local tumour were not apparent.

The addition of IL-2 to the treatment with TIL in these three patients who experienced a progression with TIL alone did not result in an objective response.

At the final analysis of this phase I trial, five patients had died from melanoma, 2–15 months after therapy, while four patients were alive with evidence of disease 8–16 months after the first infusion of TILs.

### *Immunological evaluation*

Assessment of plasmatic TNF-α, 4–7 days after each infusion, showed a maximum of a 1.2- to 22-fold increase following TIL therapy (Table III). All patients but one had the maximum level of TNF-α after the last infusion of TIL.

No change was observed in CD45RA, CD45RO, CD25, CD28, CD29W of peripheral blood cells before and after each TILs infusion.

### Discussion

Previous studies of treatment with TILs alone have reported only limited toxicities (Kradin *et al.*, 1987; Bukowski *et al.*, 1991; Marincola *et al.*, 1993), which did not include grade 3 side-effects. In our study, WHO grade 3 toxic events occurred in six patients after 13/34 infusions. Modifications in arterial blood pressure and heart rate were encountered more often in our study than in the NCI trial (21), in which no grade 3 hypotension was seen. In particular, hypotension and accelerated heart rate were more frequent when TILs were infused once a week. Following TIL therapy, a minor pericarditis was detected by a systematic echocardiography after the last infusion in two patients. In each case, the extent of pericarditis was limited and there were no clinical signs.

Compared with other studies (Kradin *et al.*, 1987; Marincola *et al.*, 1993), the observed increase in frequency and severity of these side-effects could be due to the larger number of cells infused (Kradin *et al.*, 1987) and to a longer period of follow-up after TIL infusions (Marincola *et al.*, 1993). In our study, TILs were administered over a shorter period of time (15–30 min) compared, for example, with 30–60 min infusion time in the NCI study. Considering TIL infusions without subsequent IL-2 treatment (29/34 infusions), the mean time of maximum changes in arterial blood pressure and heart rate occurred after 4.5 and 3.8 h respectively. Repeated infusion of TILs alone did not result in an objective response, although five patients presented a significant decrease in tumour volume in at least one tumour site. This response could be evaluated in three patients with subcutaneous and lymph node lesions by signs of mild inflammation or softening of tumour prior to decrease in size. In one patient with multiple subcutaneous lesions localised in one leg, there was also an objective response in conjunction with local pain without signs of inflammation. Therefore, clinical inflammation or softening of the tumour seemed to be associated with a clinical response of the subcutaneous melanoma. Histological changes with lymphocytic infiltrates, tumour necrosis and lymphocytic vasculitis have been reported in patients with subcutaneous metastases of lung carcinoma showing an objective response to immunotherapy with TILs and IL-2 (Kradin *et al.*, 1989). More recently, it was reported that the percentage of tumour necrosis present after TIL administration combined with IL-2 was significantly higher in responder than in non-responder patients with melanoma, in comparison with the pre-TIL baseline value (Cole *et al.*, 1994).

The benefit of surgical excision after a previous exposure to TILs to produce more autocytotoxic $CD8^+$ T cells in a new TIL culture cannot be assumed from the single patient case in this study. Nevertheless, there was a switch from a predominant $CD4^+$ phenotype (94%) to a $CD8^+$ population: however, no autocytotoxicity could be detected.

Whether or not TILs given to patients are able to induce cytokine secretion and thus activate immunocompetent cells remains unclear. An increase in plasmatic TNF-α could be detected after 4–7 days following TIL infusion. TNF-α is known to have a short half-life (Beutler *et al.*, 1986) and is not usually produced by unstimulated TIL *in vitro* (Schwartzentruber *et al.*, 1991; Hom *et al.*, 1993). However, recent reports have described the release of cytokines, including TNF-α, by *in vitro* co-culture of TILs with either autologous tumour stimulation (Schwartzentruber *et al.*, 1991) or, more recently, with HLA-matched melanoma tumour cells (Hom *et al.*, 1993). Secretion of TNF-α after stimulation *in vitro* followed a lag period and was maximal within the first 24 h (Schwartzentruber *et al.*, 1991). In this study, TILs given *in vivo*, and in the absence of exogenous IL-2, were able to induce levels of TNF-α detectable in the serum. At this point, we can only speculate on the nature of this TNF-α increment. Increased levels of TNF-α could be a non-specific phenomenon following infusion of a large number of immune cells; or alternatively, it could reflect a more specific interaction between immune T cells and malignant melanoma antigens.

Following repeated TIL infusions, we did not find any phenotypic changes in the peripheral blood cells. The lack of such changes may suggest that there was no activation of the cells of the immune system in this study, just as reported in a previous report for natural killer, LAK and T cells (CD45RO, CDW29) following single TIL infusion (Bukowski *et al.*, 1991).

After progression of disease under TIL treatment, the three patients infused together with IL-2 still did not present any objective response. This finding is in contrast to some (Rosenberg *et al.*, 1988; Topalian *et al.*, 1988) but not other (Dorval *et al.*, 1992) reports claiming that the combination of TIL + IL-2 can reinduce an objective response after failure of IL-2 alone.

In conclusion, repeated infusions of more than $10^{10}$ TILs are feasible, without severe side-effects. However, WHO grade 3 side-effects were more frequently observed in this study than in previous trials. No objective tumour response was observed, although some biological changes were noted, including an increase in plasmatic levels of TNF-α.

**Acknowledgements**

This work has been partially supported by Grant No. 415-93 from Association pour la Recherche sur le Cancer and by a grant from the Ligue Nationale Contre le Cancer, Comité Départemental de la Gironde.

The authors thank the nurses of the Department of Medical Oncology of Fondation Bergonié, who provided the patients with excellent care, and Dorothée Quincy and Martine Duhar for their assistance in the preparation of the manuscript.

**References**

AVRIL MF, RAVAUD A, GASPARD MH, BEYLOT C, BONNERANDI JJ, CUPISSOL D, DELAUNAY M AND TOURANI J. (1993). Treatment in metastatic melanoma with subcutaneous interleukin 2, interferon alpha 2a and decarbazine (abstract 190). *Melanoma Res.*, **3**, S3.

BALCH CM, HOUGHTON A AND PETERS L. (1989). Cutaneous melanoma. In *Cancer Principles and Practice of Oncology*, De Vita VT, Hellman S, Rosenberg SA (eds) pp. 1499–1542. Lippincott: Philadelphia.

BEUTLER BA, MILSARK IW AND LARANNI A. (1986). Cachectin tumor necrosis factor: production, distribution and metabolic fate in vivo. *J. Immunol.*, **135**, 3972–3977.

BUKOWSKI RM, SHARFMAN W, MURTHY S, RAYMAN P, TUBBS R, ALEXANDER J, BUDD GT, SERGI JS, BAUER L, GIBSON V, STANLEY J, BOYETT J, PONTES E AND FINKE J. (1991). Clinical results and characterization of tumor-infiltrating lymphocytes with or without recombinant interleukin 2 in human metastatic renal cell carcinoma. *Cancer Res.*, **51**, 4199–4205.

COLE DJ, TAUBENBERGER JK, POCKAJ BA, YANNELLI JR, CARTER C, CARRASQUILLO J, LEITMAN S, STEINBERG SM, ROSENBERG SA AND YANG YC. (1994). Histopathological analysis of metastatic melanoma deposits in patients receiving adoptive immunotherapy with tumor-infiltrating lymphocytes. *Cancer Immunol. Immunother.*, **38**, 299– 303.

DILLMAN RO, OLDHAM RK, BARTH NM, COHEN RJ, MINOR DR, BIRCH R, YANNELLI JR, MALECKAR JR, SFERRUZZA A, ARNOLD J AND WEST WH. (1991). Continuous interleukin-2 and tumor infiltrating lymphocytes as treatment of advanced melanoma. A national biotherapy study group trial. *Cancer*, **68**, 1–8.

DORVAL T, MATHIOT C, BRANDELY M, ESCANDE MC, FRIDMAN WH AND POUILLART P. (1992). Lack of effect of tumor infiltrating lymphocytes in patients with metastatic melanoma who failed to respond to interleukin 2 (letter). *Eur. J. Cancer*, **28**, 615-616.

FISHER B, PACKARD BS, READ EJ, CARRASQUILLO JA, CARTER CS, TOPALIAN SL, YANG JC, YOLLES P, LARSON SM AND ROSENBERG SA. (1989). Tumor localization of adoptively transferred indium-111 labeled tumor infiltrating lymphocytes in patients with metastatic melanoma. *J. Clin. Oncol.*, **7**, 250–261.

FOSSATI G, ANICHINI A, SQUARCINA P, MAZZOCCHI A AND PARMIANI G. (1988). Proliferative and or cytotoxic activity of lymphocyte clones to autologous human melanoma. *Int. J. Cancer*, **42**, 239–245.

HANSON J, PETIT R, WALKER M, KURTZ J, ROHLOFF C, KABLER-BABBITT C, ALEEM J AND RAUSCH C. (1991). Phase II study of tumor infiltrating lymphocytes (TIL) therapy with bolus recombinant interleukin-2 in metastatic melanoma (abstract 1035). *Proc. Am. Soc. Clin. Oncol.*, **10**, 295.

HOM SS, SCWARTZENTRUBER DJ, ROSENBERG SA AND TOPALIAN SL. (1993). Specific release of cytokines by lymphocytes infiltrating human melanomas in response to shared melanoma antigens. *J. Immunother.*, **13**, 18–30.

ITOH K, PLATSOUCAS CD AND BALCH CM. (1988). Autologous tumor-specific cytotoxic T lymphocytes in the infiltrate of human metastatic melanomas. *J. Exp. Med.*, **168**, 1419–1441.

KIRKWOOD JM AND ERNSTOFF M. (1986). Potential applications of the interferons in oncology: Lessons drawn from studies of human melanoma. *Semin. Oncol.*, **13** (Suppl. 2), 48–56.

KRADIN RL, BOYLE LA, PREFFER FI, CALLAHAN RJ, BARLAI-KOVACH M, STRAUSS HW, DUBINETT S AND KURNICK JT. (1987). Tumor-derived interleukin-2-dependent lymphocytes in adoptive immunotherapy of lung cancer. *Cancer Immunol. Immunother.*, **24**, 76–85.

KRADIN RL, KURNICK JT, LAZARUS DS, PREFFER FI, DUBINETT SM, PINTO CE, GIFFORD J, DAVIDSON E, GROVE B, CALLAHAN RJ AND STRAUSS HW. (1989). Tumour infiltrating lymphocytes and interleukin 2 in treatment of advanced cancer. *Lancet*, **i**, 577–580.

MARINCOLA FM, BALKISSON J, SCHWARTZENTRUBER DJ AND HOM SS. (1993). Hemodynamic effects of the administration of tumor infiltrating lymphocytes to cancer patients. *J. Immunother.*, **13**, 282-288.

MARKOWITZ A, PARKINSON D, ITOH K, LEGHA S, VON ESHENBACH A, LOGOTHETIS C, YEOMANS A, THIEVOLDT D, KILBOURN M, HUNTER C, GUTTERMAN J AND BALCH C. (1991). Phase I B study of tumor infiltrating lymphocytes (TIL) combined with recombinant interferon alpha 2A, recombinant interleukin 2 and cyclophosphamide in patients with advanced renal cell carcinoma and malignant melanoma (abstract 726). *Proc. Am. Soc. Clin. Oncol.*, **10**, 216.

MUUL LM, SPIESS PJ, DIRECTOR EP AND ROSENBERG SA. (1987). Identification of specific cytolytic immune responses against autologous tumor in humans bearing malignant melanoma. *J. Immunol.*, **138**, 989-995.

RAVAUD A, LAKDJA F, DELAUNAY M, COULON V, REGAUDIE JJ AND BUI BN. (1992). Cardiomyopathy following acute myocardial infarction after interleukin-2 (IL-2) and tumour infiltrating lymphocytes (TIL) therapy (letter). *Eur. J. Cancer*, **28A**, 1772.

ROSENBERG SA, PACKARD BS, AEBERSOLD PM, SOLOMON D, TOPALIAN SL, TOY ST, SIMON P, LOTZE MT, YANG JC, SEIPP CA, SIMPSON C, CARTER C, BOCK S, SCHWARTZENTRUBER D, WEI JP AND WHITE DE. (1988). Use of tumor infiltrating lymphocytes melanoma and interleukin 2 in the immunotherapy of patients with metastatic melanoma. *N. Engl. J. Med.*, **319**, 1676-1681.

ROSENBERG SA, LOTZE MT, YANG JC, AEBERSOLD PM, LINEHAN WM, SEIPP CA AND WHITE DE. (1989). Experience with the use of high-dose interleukin-2 in the treatment of 652 cancer patients. *Ann. Surg.*, **210**, 474-485.

SCHWARTZENTRUBER DJ, TOPALIAN SL, MANCINI M AND ROSENBERG SA. (1991). Specific release of granulocyte-macrophage colony-stimulating factor, tumor necrosis factor-α, and IFN-γ by human tumor-infiltrating lymphocytes after autologous tumor stimulation. *J. Immunol.*, **146**, 3674-3681.

SPIESS PJ, YANG JC AND ROSENBERG SA. (1987). *In vitro* antitumor activity of tumor-infiltrating lymphocytes expanded in recombinant interleukin-2. *J. Natl Cancer Inst.*, **79**, 1067-1075.

TOPALIAN SL, MUUL LM, SOLOMON D AND ROSENBERG SA. (1987). Expansion of human tumor infiltrating lymphocytes for use in immunotherapy trials. *J. Immunol. Methods*, **102**, 127-141.

TOPALIAN SL, SOLOMON D, AVIS FP, CHANG AE, FREERKSEN DL, LIHEHAN WM, LOTZE MT, ROBERTSON CN, SEIPP CA, SIMON P, SIMPSON CG AND ROSENBERG SA. (1988). Immunotherapy of patients with advanced cancer using tumor infiltrating lymphocytes and recombinant interleukin 2: a pilot study. *J. Clin. Oncol.*, **6**, 839-853.

TOPALIAN SL, SOLOMON D AND ROSENBERG SA. (1989). Tumor-specific cytolysis by lymphocytes infiltrating human melanomas. *J. Immunol.*, **142**, 3714-3725.